# Photothermal Effect and Multi-Modality Imaging of Up-Conversion Nanomaterial Doped with Gold Nanoparticles

**DOI:** 10.3390/ijms23031382

**Published:** 2022-01-26

**Authors:** Wei Zhang, Yang Zang, Yanli Lu, Jinhui Han, Qingyun Xiong, Jinping Xiong

**Affiliations:** 1 Beijing Key Laboratory of Electrochemical Process and Technology of Materials, Beijing University of Chemical Technology, Beijing 100029, China; 2019200387@mail.buct.edu.cn (W.Z.); 2019310030@mail.buct.edu.cn (Y.Z.); 2019310036@mail.buct.edu.cn (Y.L.); 2020200494@mail.buct.edu.cn (Q.X.); 2 State Key Laboratory of Organic-Inorganic Composites, Beijing University of Chemical Technology, Beijing 100029, China; 2021400008@mail.buct.edu.cn; 3 College of Ecology and Resources Engineering, Wuyi University, Jiangmen 354300, China

**Keywords:** up-conversion, nanomaterials, photothermal, multi-modality imaging

## Abstract

Two key concerns exist in contemporary cancer chemotherapy in clinics: limited therapeutic efficiency and substantial side effects in patients. In recent years, researchers have been investigating revolutionary cancer treatment techniques and photo-thermal therapy (PTT) has been proposed by many scholars. A drug for photothermal cancer treatment was synthesized using the hydrothermal method, which has a high light-to-heat conversion efficiency. It may also be utilized as a clear multi-modality bioimaging platform for photoacoustic imaging (PAI), computed tomography (CT), and magnetic resonance imaging (MRI). When compared to single-modality imaging, multi-modality imaging delivers far more thorough and precise details for cancer diagnosis. Furthermore, gold-doped upconverting nanoparticles (UCNPs) have an exceptionally high target recognition for tumor cells. The gold-doped UCNPs, in particular, are non-toxic to normal tissues, endowing the as-prepared medications with outstanding therapeutic efficacy but exceptionally low side effects. These findings may encourage the creation of fresh effective imaging-guided approaches to meet the goal of photothermal cancer therapy.

## 1. Introduction

Interest in developing theranostic nanoplatforms with simultaneous diagnostic and therapeutic capacity has gradually increased in the nanomedicine field because it provides significant prospects in the treatment of major illnesses including cancer [1]. Imaging probes, as one of the most important components of the theranostic nanoplate-form, should be able to perform many levels of imaging at the same time, from the cell to the whole body to offer comprehensive tumor characteristics for clinical diagnostics. However, single-modality imaging did not match the high diagnostic criteria since each imaging technique (optical imaging, CT, and MRI) has intrinsic flaws due to restricted resolution, sensitivity, or imaging depths.

To mitigate this problem, several imaging probes were combined into a single multi-modality imaging system, even with some considerable restrictions such as sophisticated synthetic processes and heterogeneous nanostructures. As a result of their enhanced optical and magnetic properties, and also improved X-ray attenuation, lanthanide-doped upconverting nanoparticles (UCNPs) might be perfect for building multifunctional bio probes by doping with various rare earth ions without modifying other functions.

Many researchers have recently advocated that it be employed in biological imaging since it provides considerable benefits in the treatment against major illnesses such as cancer. However, the typical challenge is that it has insufficient light intensity and is poisonous to cells, therefore its structure and surface must be modified [2]. Many scholars have proposed doping Mo^3+^, Cu^2+^ [3,4], and other metal ions in the NaYF_4_:Yb^3+^/Er^3+^ unit cell to increase the luminous intensity [5,6], but the effect is not significant. Others have offered sliver doping [7], which has a large impact as well, however sliver is poisonous to cells, may cause cell death without targeting, and cannot be employed in biology. Many scholars have proposed constructing core-shell structures such as NaYF_4_:Yb^3+^/Er^3+^@NaGdF_4_:Yb^3+^ and NaYF_4_:Yb^3+^/Er^3+^@NaNdF_4_:Yb^3+^/Tm^3+^@NaGdF_4_:Yb^3+^ [8,9,10] or alternatively, using the reverse microemulsion method to construct a layer of silica or porous silica, such as NaYF_4_:Yb^3+^/Er^3+^@SiO_2_, NaYF_4_:Yb^3+^/Er^3+^@NaGdF_4_:Yb^3+^@m-SiO_2_ [11,12,13,14]. Gold nanoparticles are currently the mainstream biomaterials in tumor diagnosis and treatment applications [15,16]. Gold nanoparticles are widely used in CT imaging and photoacoustic imaging due to their excellent imaging capabilities and photothermal effects [17]. However, in our research, their photothermal stability seems to be not very good [18]. Unfortunately, when these materials meet the biological requirements, they will inevitably reduce their luminous intensity, so that imaging cannot be performed to obtain a clear image [19,20]. Considering the high desire to develop UCNPs nanomaterials with highly effective imaging capability as well as high biocompatibility to prevent apoptosis or biological organ failure, UCNPs doped with gold nanoparticles are an ideal candidate because they are easy to fabricate, have enhanced luminescence, and are easy to surface modify [21,22]. More notably, following illumination, the UCNPs is harmless to normal tissues but cytotoxic to malignancies. To the best of our knowledge, however, there appears to be a failure in the literature to yet create theranostic nanoplatforms integrating multi-modality bioimaging with light trigger chemotherapy.

## 2. Results and Discussion

The TEM images of gold nanoparticles (Figure S1) prepared using the hydrothermal method show that they are spherical and have an average diameter of 5 nm. TEM images show the morphology of Au-UCNPs (Figure S2). They are rod structures with a length of 50 nm–100 nm and narrow ends. The reason for this phenomenon is that during the nucleation and growth of the nanoparticles by the coprecipitation-hydrothermal method, the temperature controls its width and shape, and the time determines its length. When the temperature starts to drop, the two ends of the nanorod begin to shrink with the decrease of temperature, and finally show the phenomenon of narrowing at both ends. The successful doping of gold nanoparticles into UCNPs was proved by energy spectrum (Figure S3).

**Figure 1:**
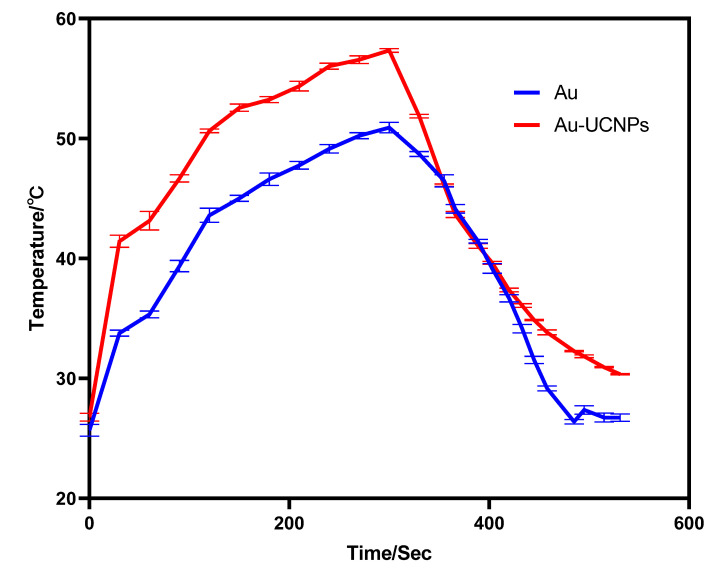
Temperature rise and temperature drop curves of gold nanoparticles (blue) irradiated by near-infrared light at 540 nm wavelength, and Au-UCNPs (red) irradiated by near-infrared light at 980 nm wavelength.

**Figure 2:**
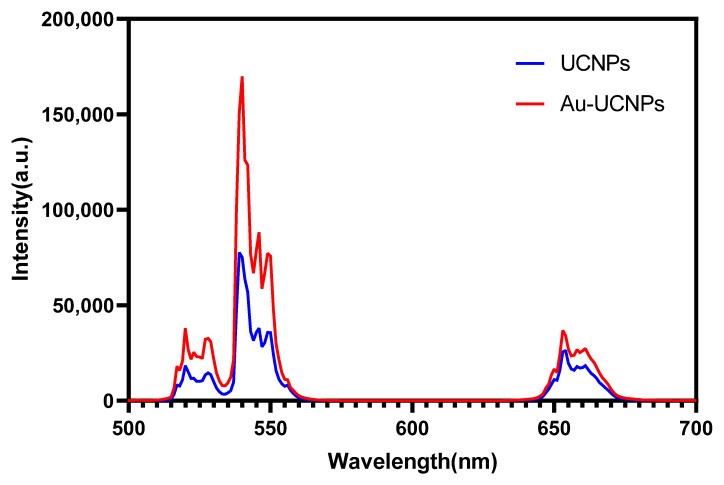
Luminescent intensity of UCNPs (blue) and Au-UCNPs (red).

**Figure 3:**
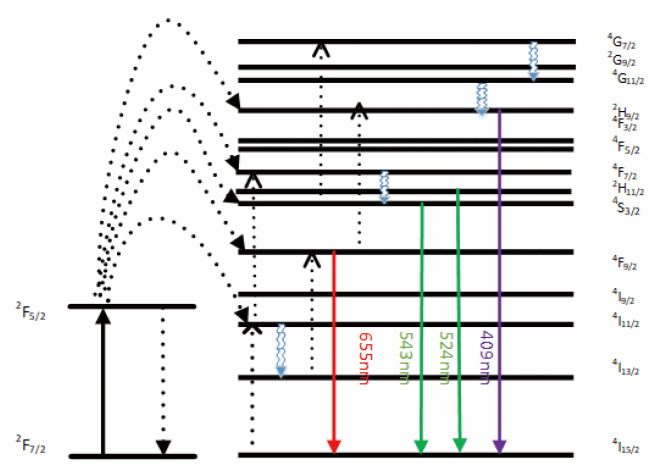
A simplified energy level diagram of Er^3+^/Yb^3+^ system doped with Au and up-conversion pathways.

The gold nanoparticles exhibit a temperature increase phenomenon when exposed to a laser with a wavelength of 540 nm and a power of 500 mW (Figure 1). The irradiation ends when the temperature reaches 52 °C, and the gold nanoparticles begin to cool. When the temperature lowers to room temperature, the gold nanoparticles are bombarded with a laser once again, and they no longer heat up. However, when gold nanoparticles are doped into UCNPs that have already been irradiated by a laser with a wavelength of 980 nm and a power of 500 mW. At the same time, Au-UCNPs are heated to 57 °C and immediately cooled once the irradiation is stopped. It is commonly known that near-infrared light irradiation at 980nm wavelength, UCNPs can only generate green visible light without producing heat. However, after doping gold nanoparticles, it can not only emit brighter visible light as emitting before doping the gold nanoparticles (Figure 2) but also emit heat. The reasons for this phenomenon are as follows:

The emission bands are observed at 409 nm (purple), 524 nm, 543 nm (green), and 655 nm (red) are due to the transitions of Er^3+^ ions such as ^2^H_9/2_ → ^4^I1_5/2_, ^2^H_11/2_ → ^4^I_15/2_, ^4^S_3/2_ → ^4^I_15/2_ and ^4^F_9/2_ → ^4^I_15/2_, respectively (Figure 3). However, it is observed that the red and green emissions become more prominent than the purple emission, which might be attributed to the cross-relaxation process from ^2^G_7/2_ to ^2^H_9/2_ levels. Purple, green, and red lights exhibit varying degrees of enhancement. The SPR absorption peak at 520 nm coincides with the emission band of green light, so the SPR vibration frequency overlaps the luminescence band of UCNPs, the coupling of the emitted light and the SPR increases the photon localized state density near the surface of Au, thereby increasing the radiation decay rate of Er^3+^, also increasing the luminous intensity. Moreover, the excitation wavelength of 980 nm excites the higher-lying ^4^F_7/2_ level of Er^3+^ ions and Au partially absorb emissions coming from Er^3+^ ions that lead to de-excitation of the fluorescence of UCNPs.

Simultaneously, gold nanoparticles enhance the luminescence intensity of UCNPs under the near-infrared light with the wavelength of 980 nm, so that UCNPs emit stronger light energy with the wavelength of 540 nm. This light energy further excites gold nanoparticles, resulting in the heat emission of UCNPs doped with gold nanoparticles [23].

**Figure 4:**
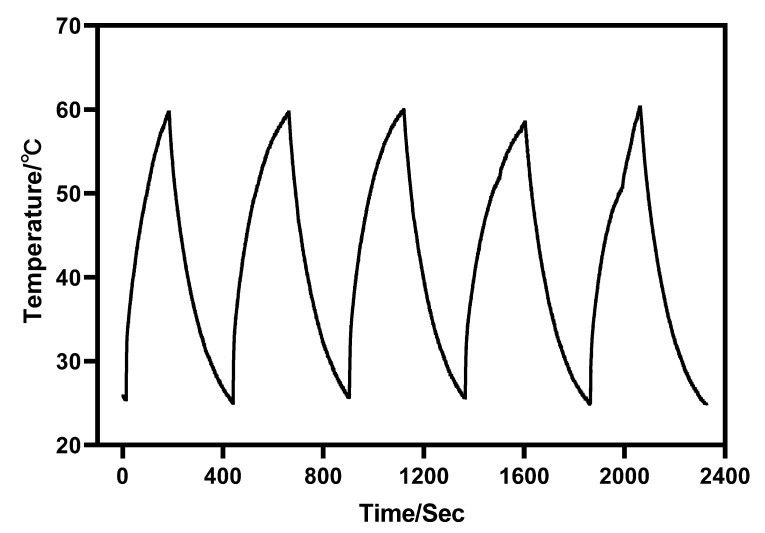
Temperature rise and temperature drop cycle curve of 180 µg/mL of Au-UCNPs (5 times).

Furthermore, under the irradiation of near-infrared light with a wavelength of 980 nm, Au-UCNPs raises the temperature to 59 °C in 250 s, immediately stops the irradiation, cools to room temperature in about 160 s, re irradiates for about 280 s, raises the temperature to 59 °C again, and repeats the cycle five times. Au-UCNPs still show good photothermal effect (Figure 4). However, gold nanoparticles cannot be heated and cooled again under 540 nm wavelength laser irradiation (Figure 1), which shows that gold nanoparticles do not have good photothermal stability (Figure S4). Many experts [24] think that when gold nanoparticles are not modified in any way, the outer electrons of gold nanoparticles are stimulated in the excited state, and the energy level transition happens during the process of returning to the ground state. The surface structure of gold nanoparticles has altered during the energy conversion process, resulting in a mild exothermic effect when re-excited (Figure S5).

A displacement solid solution is generated when Au nanoparticles are doped into UCNPs. Gold nanoparticles are uniformly dispersed in the hexagonal crystal structure of UCNPs, replacing some of the Er. After 980 nm near-infrared light stimulation, Au and Er contribute jointly to the hybrid orbit, enhancing the electron transition, carrier concentration, and luminescence of UCNPs. As a result, the intensity of the excitation light source of gold nanoparticles rises, thereby improving their photothermal conversion capacity. Particularly, the outer electrons of gold nanoparticles are in the excited state, and some Au nanoparticles provide hybrid orbits. Therefore, the surface structure of gold nanoparticles will hardly change, which greatly enhances the photothermal stability of gold nanoparticles.

**Figure 5:**
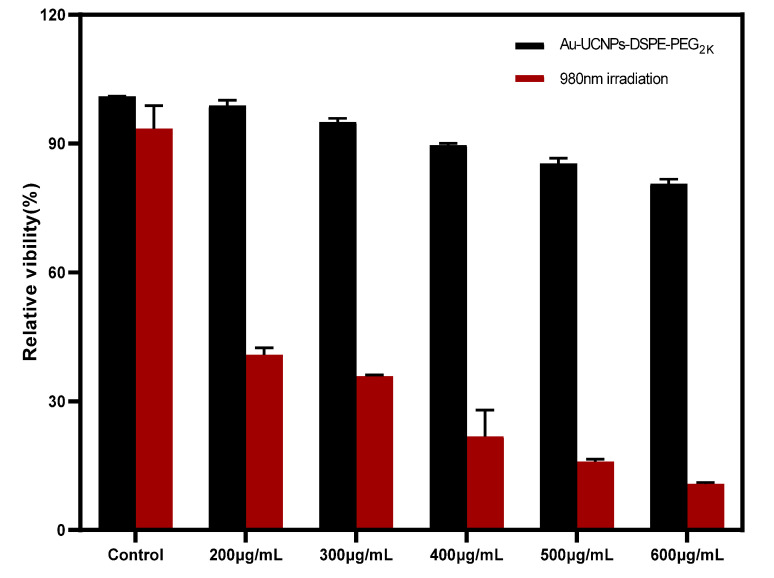
Cytotoxicity of different concentrations of Au-UCNPs-DSPE-PEG_2__K_ before and after irradiation.

The cytotoxicity of Au-DSPE-PEG_2K_ is analyzed by microplate reader. HeLa cells are cultivated for 4 h after dispersing modified rare-earth nanomaterials in normal saline to prepare various quantities, and their activity is assessed (Figure 5). The cell survival rate is greater than 89% at the concentration of Au-UCNPs-DSPE-PEG_2K_ is less than 400 µg/mL. Particularly, at 200 µg/mL, the cell survival rate is greater than 99%. According to Figure 2, a 200 µg/mL concentration of rare-earth nanoparticles not only has appropriate safety but also has a high luminous intensity. Even when the rare-earth ion concentration is as high as 500 or 600 µg/mL, cell survival remains greater than 80%.

UCL imaging (Figure S6) yield clear cells makers at an excitation wavelength of 980 nm, clear cells markers were obtained. The figure shows that after 5 min of incubation, there is light source signal on the cells, and after 2 h even 6 h, the light source signal still exists indicating that the sample that was easily absorbed by the Hela cells and can last for a long time. A 200 ug/mL concentration of Au-UCNPs-DSPE-PEG_2K_ was injected intratumorally into mice. In vivo imaging (Figure S7) yielded clear tumor makers at an excitation wavelength of 980 nm and clear tumor markers were obtained. The figure shows that after 180 min of incubation, there is no other light source signal besides the tumor, indicating that the sample that was injected into the body has no outflow.

**Figure 6:**
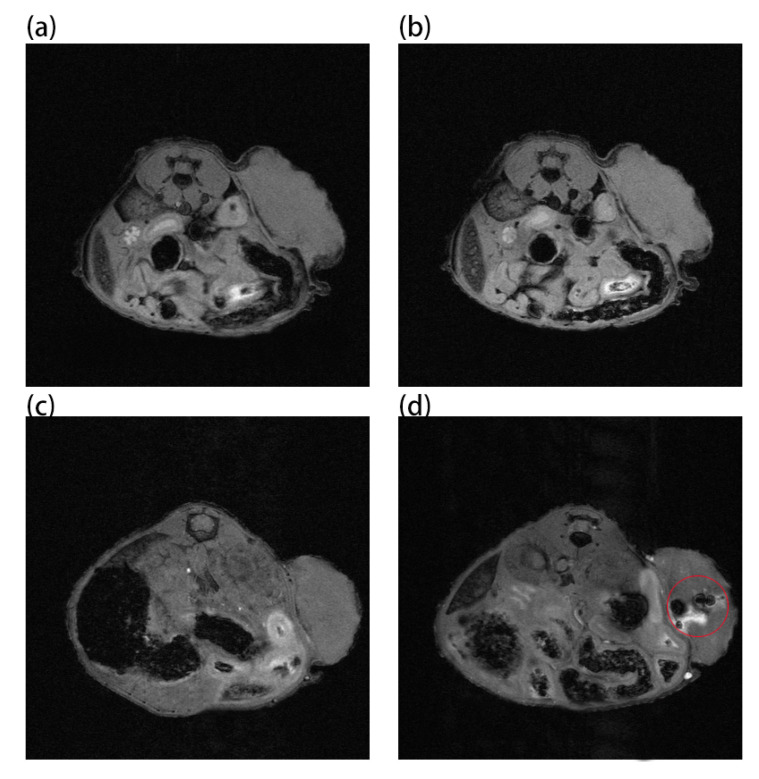
MR images before and after intratumor injection of Au and Au-UCNPs-DSPE-PEG_2__K_ in Balb/c mice, (**a**,**b**) are before and after injection of Au, (**c**,**d**) are before and after injection of Au-UCNPs-DSPE-PEG_2__K_.

Au and Au-UCNPs-DSPE-PEG_2K_ nanoparticles are injected intravenously into Balb/c mice (Figure 6). As can be seen from Figure 6a,b, there is no difference in MRI images before and after gold nanoparticles injection, demonstrating that gold nanoparticles have no MRI imaging capabilities due to their lack of X-ray attenuation. After injection of Au-UCNPs nanoparticles, obvious MRI signals appear at the tumor location (in the red circle in Figure 6d), which is due to the X-ray attenuation characteristics of UCNPs (Figure S8).

**Figure 7:**
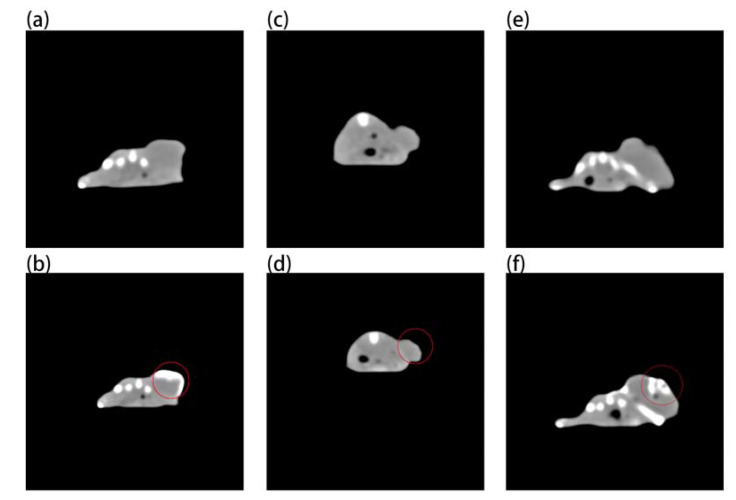
Micro-CT images before and after intratumor injection of Au and Au-UCNPs-DSPE-PEG_2__K_ in Balb/c mice, (**a**,**b**) are before and after injection of high concentration (50 mg/mL) of Au, (**c**,**d**) are before and after injection of low concentration (180 µg/mL) of Au, (**e**,**f**) are before and after injection of 180 µg/mL of Au-UCNPs-DSPE-PEG_2__K_.

Figure 7 shows that although gold nanoparticles have imaging ability (Figure 7b), they need a very high concentration, while Au-UCNPs need a low concentration. This is why a very obvious CT signal is detected (Figure 7f). Gold nanoparticles and Au-UCNPs are at the same concentration, and Au cannot observe the CT signal (Figure 7d).

**Figure 8:**
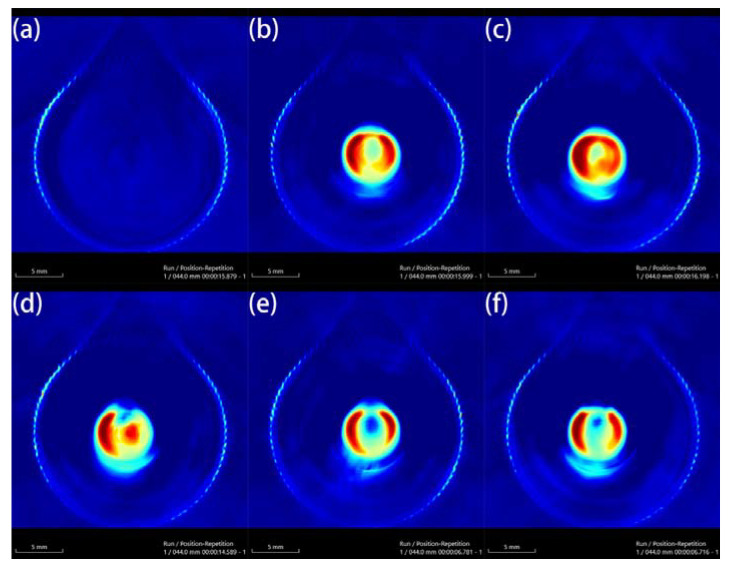
Photoacoustic imaging (PAI) of different concentrations of Au-UCNPs-DSPE-PEG_2K_, (**a**) 0 µg/mL, (**b**) 60 µg/mL, (**c**) 120 µg/mL, (**d**) 180 µg/mL, (**e**) 240 µg/mL, (**f**) 300 µg/mL.

**Figure 9:**
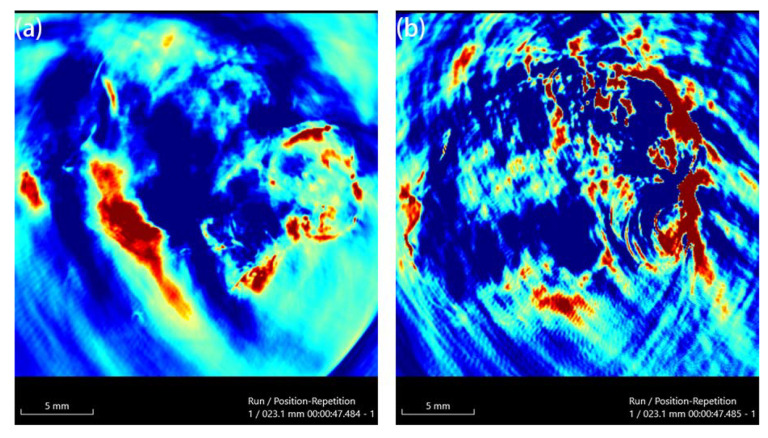
PAI of before (**a**) and after (**b**) injection 180 µg/mL of Au-UCNPs-DSPE-PEG2K in Balb/c mice.

Au-UCNPs-DSPE-PEG_2K_ has excellent photoacoustic properties because of its excellent photothermal effect, which are characterized by photoacoustic imaging (Figure 8), and strong photoacoustic signals can be observed. Figure 9 shows that when injection the concentration of Au-UCNPs-DSPE-PEG_2K_ is 180 µg/mL, PA value is very obvious.

## 3. Material and Method

### 3.1. Applied Chemicals

Y_2_O_3_ (99.99%), Yb_2_O_3_ (99.99%), Er_2_O_3_ (99.99%), nitric acid (68%), sodium fluoride (99.99%), citric acid (99.99%), cyclohexane (99.5%), Chlorauric acid (99.99%), ethylenediaminetetraacetic acid (EDTA, ≥99%), sodium hydroxide (≥98%) and polyethylene pyrrolidone (PVP, average molecular weight of 1,000,000–1,500,000) were purchased from Aladdin. The cell counting kit 8 (CCK-8) assay kit was purchased from BOVOGEN. All chemicals were used as received without additional purification.

### 3.2. Synthesis of Nanomaterials

#### 3.2.1. Synthesis of Au Nanoparticles

By dropping, 60 mL of 0.05 mol/L of citric acid solution was added to 3 mL of 0.02 mol/L of HAuCl_4_ to obtain a mixture. Moreover, after 5 min of continuous stirring, the solution was transferred to a 100 mL reactor and placed in an oven for the reaction at 180 °C for 12 h. The reaction was then cooled to room temperature, washed, and centrifuged to obtain solid Au nanoparticles, and then added to 10 mL of deionized water and PVP, and then placed in a test tube to prepare the sol for use.

#### 3.2.2. Synthesis of Au-UCNPs

RE_2_O_3_ (RE = Y, Yb, Er) was heated to achieve complete dissolution in excess nitric acid and then transferred to a vacuum system for evaporation to obtain a solid RE(NO_3_)_3_, which was then dissolved in deionized water and recrystallized twice. A certain amount of solid RE(NO_3_)_3_ was dissolved in deionized water, and EDTA (molar ratio of EDTA: RE(NO_3_)_3_ = 1:1) was added and stirred at 600 rpm for 1 h. The mixture was then weighed and dissolved in sodium fluoride in deionized water by ultrasound, and then the solution was added and stirred at 600 rpm for 1 h. Finally, the pH value was adjusted to 5.5 with NaOH and add 10 mL of gold solution, and then place it in a hydrothermal kettle to react at 190 °C for 24 h. The reaction products were cooled, centrifuged, and washed twice with ethanol/deionized water (1:1 *v/v*), and dried in vacuum at 80 °C for 3 h. The resultant powder is dispersed in cyclohexane for later use.

#### 3.2.3. DSPE-PEG_2K_ Modified Au-UCNPs

First, 6 mL of Au-UCNPs (0.4 mmol) dispersed in chloroform were mixed with 20 mL of DSPE-PEG_2K_ (100 mg) chloroform solution in 5 mL open glass bottles. After heating at 75 °C for 5 min to remove the chloroform, adding 24 mL of water to complete the ultrasonic dispersion, stirring at 75 °C for 10 min, cooling to room temperature, centrifuging at 18,000 rpm for 8 min to take the precipitate, and adding 1 mL of normal saline to disperse, large particles were removed by centrifugation at 5000 rpm for 5 min and then dried by a blast at 75 °C.

### 3.3. Characterization

Transmission electron microscopy (TEM) measurements were performed on a JEOL 2011 microscope operating at 200 kV. All samples were first dispersed in ethanol and then collected using a Cu grid covered with a carbon film for measurement. To determine the elemental composition of the samples, energy-dispersive X-ray spectroscopy (EDS) of the samples was performed on a JEOL 2010 EDS instrument using high-resolution transmission electron microscopy (HRTEM) measurements. Inductively coupled plasma-atomic emission spectrometry (ICPAES) was performed using a Perkin Elmer 7300DV apparatus. Scanning electron microscopy (SEM) images were obtained using a Philips XL30 electron microscope operating at 20 kV. Before this characterization, an Au film was sprayed on the sample. The upconversion luminescence spectrum was obtained using a spectrum analyzer (ANDO AQ6317, Yokohama, Japan). The sample was placed in a 1.0-cm path length support, which was excited using a 980-nm CW semiconductor diode laser (Pmax 800 mW, 1000 mA). The upconversion luminescence spectrum was obtained by the spectrophotometer using a multimode fiber having a core diameter of 0.6 mm. The distance between the top of the fiber and sample is ~2 mm. A spectrum analyzer (ANDO AQ6317, Yokohama, Japan) was used to get the up-conversion luminescence spectra. The specimen was positioned in a 1.0 cm path length support and excited by utilizing a 980 nm CW semiconductor diode laser (Pmax 800 mW, 1000 mA). The up-conversion luminescence spectrum was acquired through the spectrophotometer having a multimode fiber with a core diameter of 0.6 mm. The top of the fiber was ~2 mm away from the specimen. A thermal imager (FOCUS 280DS) was used to characterize the photoacoustic properties of photographic materials. HORIBA laser and power density meter are used to characterize photothermal properties.

### 3.4. CCK-8 Assay for Cytotoxicity

HeLa cells were cultured in the logarithmic growth phase, and the culture medium was sucked out from the flask. The cells were then washed with PBS and digested with the help of 0.25% trypsin. Then, the trypsin was removed, and the cells were blown with DMEM media containing 10% fetal bovine serum before being shifted to the sample tank and blown well. Following that, 100 µL cells were introduced onto a 96-well plate (1 × 10^4^ cells/well) and cultured for 24 h at 37 °C in a constant temperature incubator (5% CO_2_) and repeated as a set. The cells of two 96-well plates were cultured in an incubator at 37 °C with 5% CO_2_ for 1.5 h at concentrations of 200, 300, 400, 500, and 600 µg/mL of Au-UCNPs-DSPE-PEG_2K_ respectively. One group was irradiated with a 980 nm wavelength and 500 mv power laser for 5 min. The culture media was cleaned separately, PBS was washed twice, and the culture medium in the 96-well plates was replaced with 100 μL of fresh DMEM containing 10% fetal bovine serum, followed by 10 μL of CCK-8 solution in each well. After 2 h of incubation, the absorbance of each well at 450 nm was measured with a microplate reader.

### 3.5. Establishment of Animal Tumor Model

A BALB/c female white mouse with SPF grade weighing 18 g was depilated, and log phase Hela cells were subcutaneously injected into the mice’s upper right hind leg to create a mouse Hela subcutaneous tumor development model. Thirty-nine SPF-grade female mice were used for tumor model construction. MRI required two control groups and two experimental groups, with 3 tumor mice in each group, a total of 12. CT imaging requires 3 groups of control group and 3 groups of treatment group, with 3 mice in each group, a total of 18 mice. PAI needs a control group and a treatment group, with 3 rats in each group, a total of 6 rats. UCL imaging needs one group, a total of 3 rats.

### 3.6. In Vitro and In Vivo UCL Imaging

For UCL imaging, HeLa cells were plated on 14 mm glass coverslips under 100% humidity and allowed to adhere for 24 h. The cells were stained with 200 μg/mL Au-UCNPs-DSPE-PEG_2K_ at 37 °C for 4 h. Prior to imaging, the coverslips were washed twice with PBS in order to remove excess Au-UCNPs-DSPE-PEG_2K_. UCL imaging of cells was performed with a modified inverted fluorescence microscopy equipped with a continuous-wave (CW) laser at 980 nm (300 mW).

The tumors were established by subcutaneous injection of Hela cells (murine hepatocarcinoma cell lines) in the right leg of each mouse. The tumors were allowed to grow for around 4 days to reach the size of around 100–200 mm^3^. The Balb/c mouse was anesthetized (with 10% chloral hydrate 100 μL) and was intratumorally injected with the Au-UCNPs-DSPE-PEG_2K_. Subsequently, the mouse was imaged by modified in vivo Maestro whole-body imaging system with an external 980 nm laser as the excitation source.

### 3.7. MRI and CT Imaging of Mice

To acquire preimages, anesthetized the mice with isoflurane during the procedure, placed them in an animal MRI scanner (NM42-040H-I) with a magnetic field strength of 1T, and executed a tomographic scan of the tumor location on mice whose tumor developed to 100 mm^3^. Then 200 µg/mL of Au-UCNPs-DESP-PEG_2K_ solution was injected, and images were collected again. Similarly, placed the mouse before and after the injection of 200 µg/mL of Au-UCNPs-DESP-PEG_2K_ solution on a SPECT/CT (tube current: 615 μA, tube voltage: 55 kV) animal bed, performed a preimage acquisition of full-angle CT imaging in precise mode.

## 4. Conclusions

An Au-UCNPs-DSPE-PEG_2K_ multi-modality bioimaging device has been finally developed and may be utilized for photothermal treatment. The combined PA imaging with CT and MRI experiments show that Au-UCNPs-DSPE-PEG_2K_ may be used as contrast mediators for tri-modal imaging for both in vitro and in vivo testing, giving complete details for tumor diagnosis. Particularly, Au-UCNPs-DSPE-PEG_2K_ has better photothermal stability than gold nanoparticles and can well repeat the heating and cooling process to achieve the purpose of tumor treatment. These nanomaterials have exhibited low cytotoxicity, indicating their high biocompatibility for an organism. All these promising findings make Au-UCNPs-DSPE-PEG_2K_ nanocomposites an auspicious candidate for cancer theranostics, and it has encouraged us to develop the integration of diagnosis and treatment of tumors.

## Data Availability

No new data were created or analyzed in this study. Data sharing is not applicable to this article.

## Supplementary Materials

The following supporting information can be downloaded at: https://www.mdpi.com/article/10.3390/ijms23031382/s1.
