# Supplementary material for: Photothermal Effect and Multi-Modality Imaging of Up-Conversion Nanomaterial Doped with Gold Nanoparticles
Source: Int J Mol Sci. 2022 Jan 26;23(3):1382. doi: 10.3390/ijms23031382 (PMC8835931; doi:10.3390/ijms23031382)
Supplement: Supplementary file 1 [file ijms-23-01382-s001.zip › ijms-1564183-supplementary.pdf]

Supplementary materials

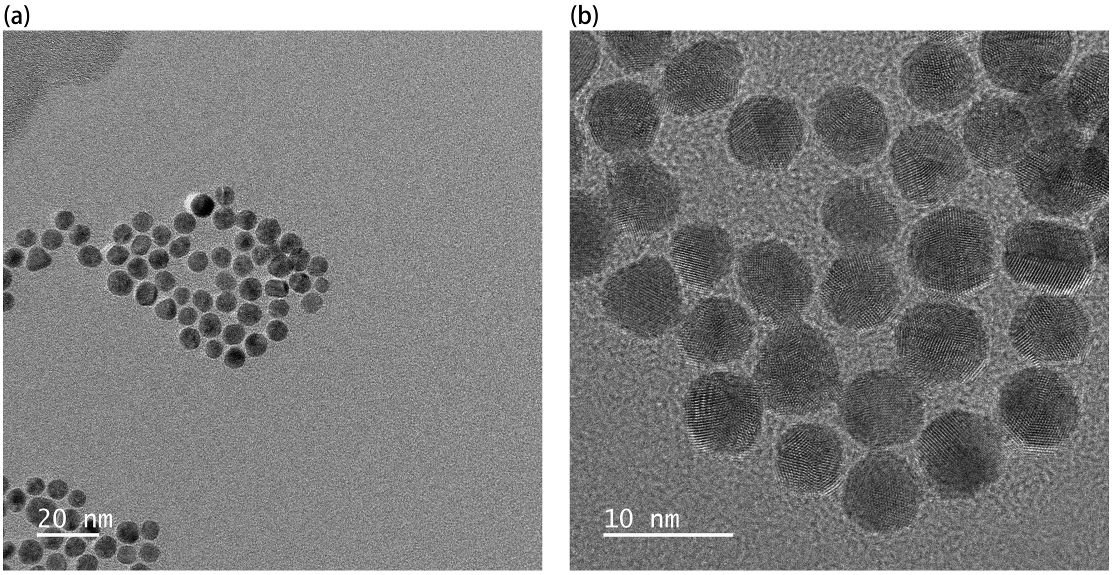

Figure S1. (a): TEM image of Au nanoparticles and (b): lattice of (a)

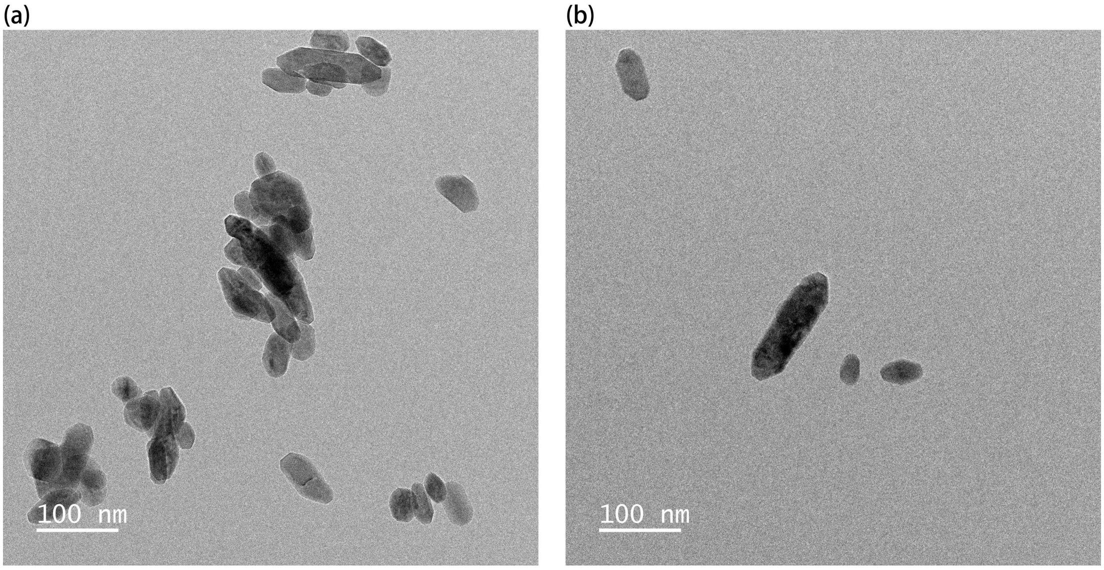

Figure S2. TEM image of Au-UCNPs

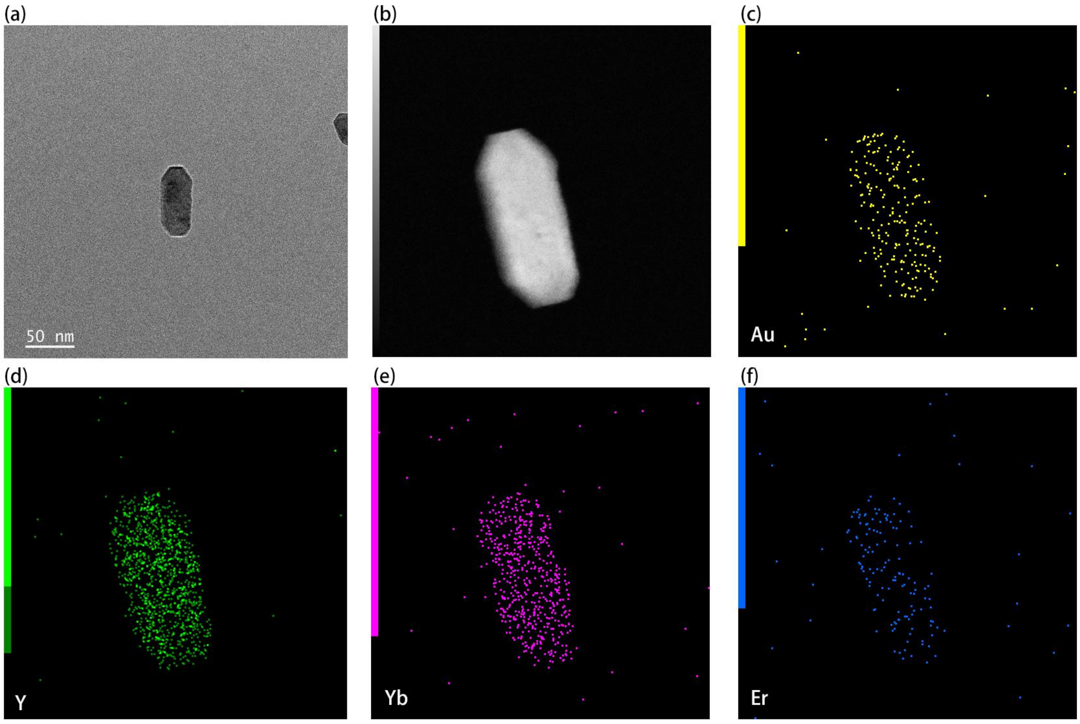

Figure S3. (a): TEM image of Au-UCNPs, (b): energy of (a), (c-f):mapping of (b)

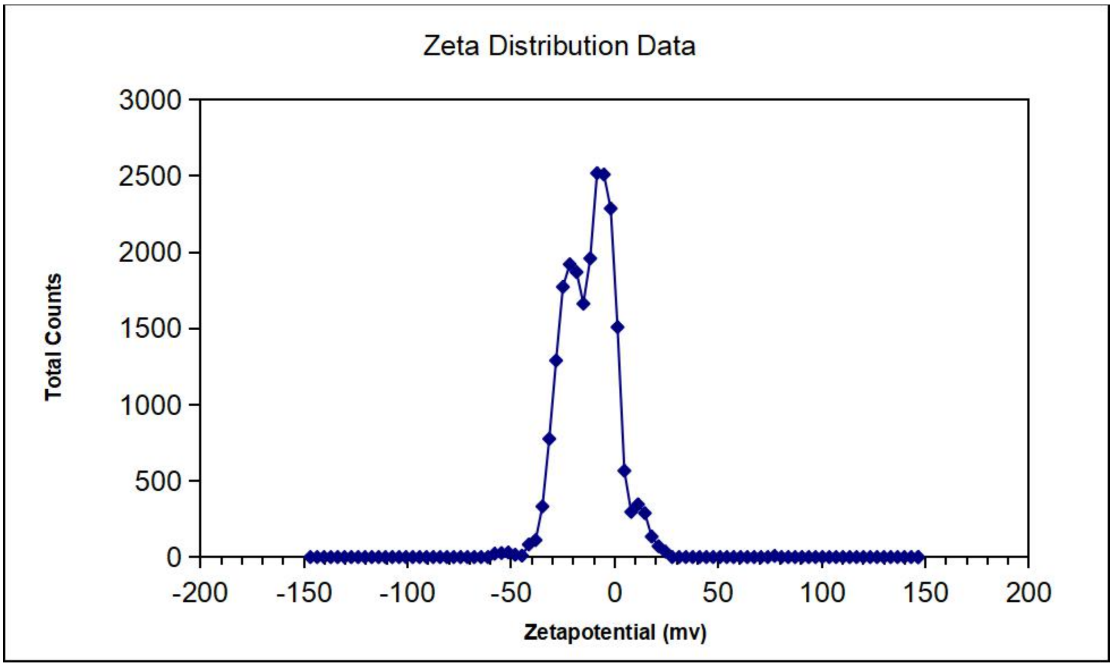

Figure S4. Zeta potential of gold nanoparticles

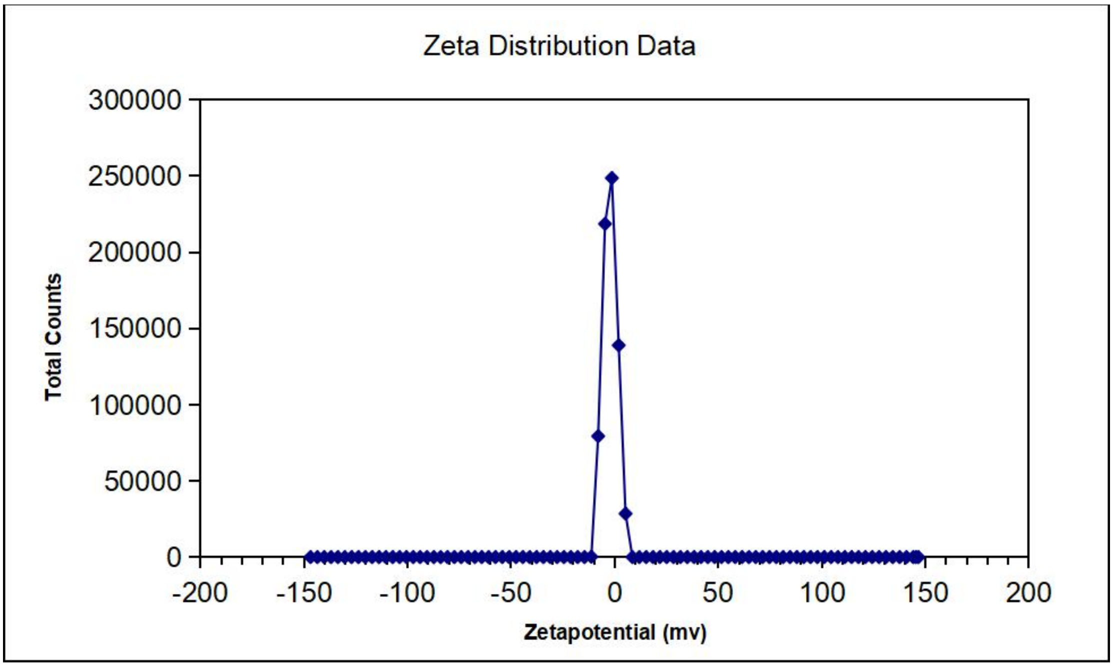

Figure S5. Zeta potential of Au-UCNPs

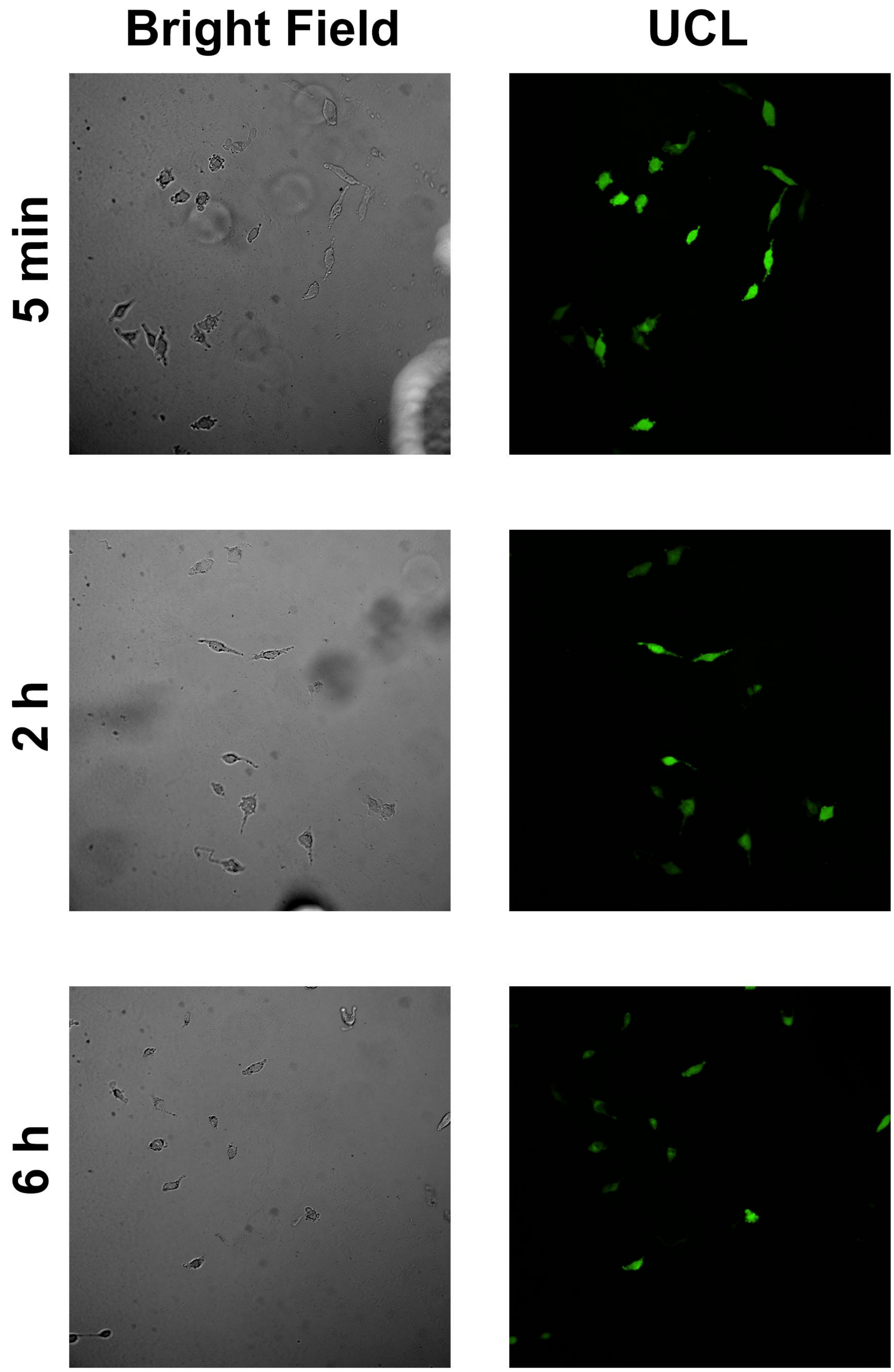

Figure S6. UCL images of HeLa cells incubated with Au-UCNPs-DSPE-PEG_{2K} for 5 min, 2 h and 6 h.

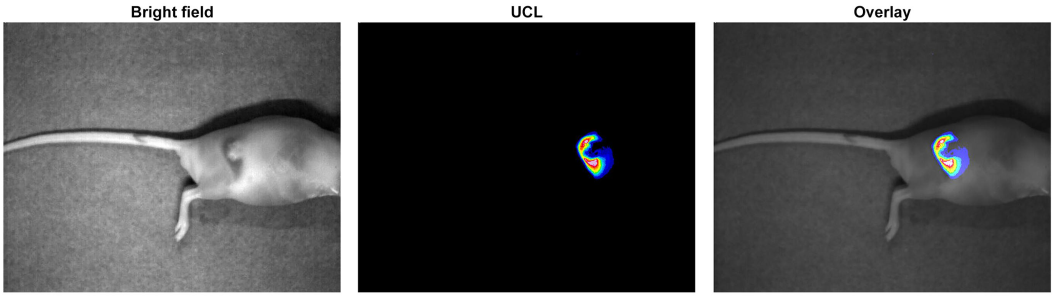

Figure S7. In-vivo imaging of a tumor-bearing Balb/c mouse after injection of Au-UCNPs-DSPE-PEG_{2K} at the tumor site

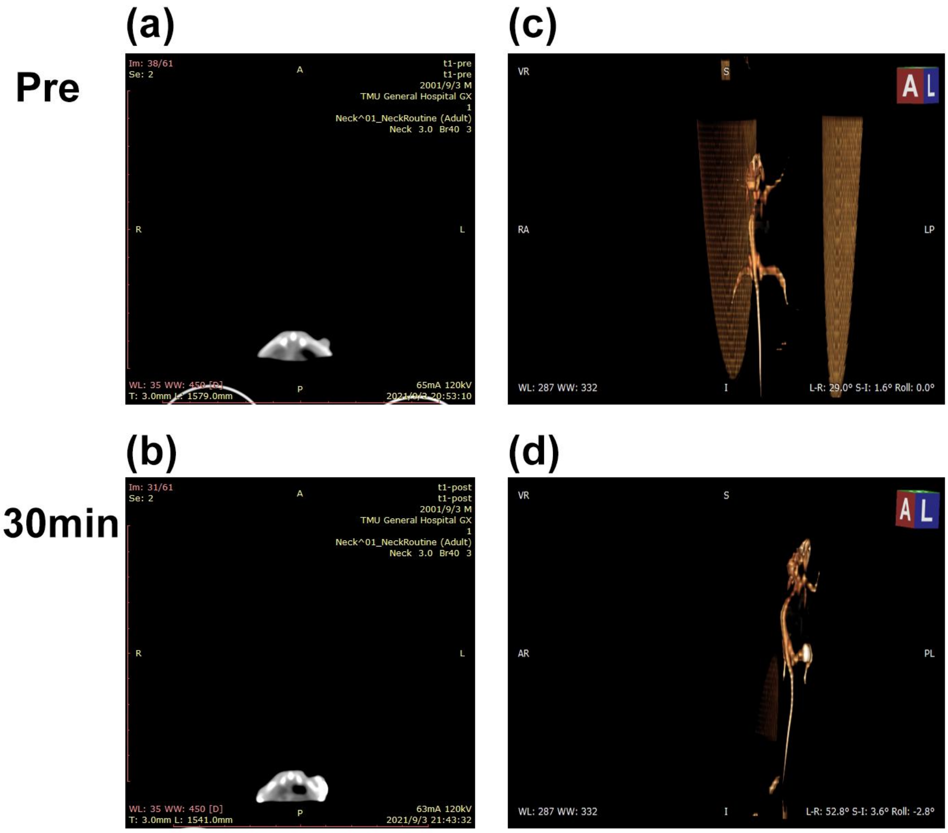

Figure S8. Micro-CT images before(a) and after(b) intratumor injection of Au-UCNPs-DSPE-PEG_{2K} in Balb/c mice, (c),(d) is the 3D model of (a) and (b) respectively
